# Improving Recognition of Defective Epoxy Images in Integrated Circuit Manufacturing by Data Augmentation

**DOI:** 10.3390/s24030738

**Published:** 2024-01-23

**Authors:** Lamia Alam, Nasser Kehtarnavaz

**Affiliations:** Department of Electrical and Computer Engineering, University of Texas at Dallas, Richardson, TX 75080, USA; lamia.alam@utdallas.edu

**Keywords:** vision-based inspection in IC manufacturing, epoxy drop images for die attachment, data augmentation via enhanced CycleGAN, supervised and unsupervised recognition of defective epoxy drop images, impact of data augmentation on recognition accuracies

## Abstract

This paper discusses the problem of recognizing defective epoxy drop images for the purpose of performing vision-based die attachment inspection in integrated circuit (IC) manufacturing based on deep neural networks. Two supervised and two unsupervised recognition models are considered. The supervised models examined are an autoencoder (AE) network together with a multi-layer perceptron network (MLP) and a VGG16 network, while the unsupervised models examined are an autoencoder (AE) network together with k-means clustering and a VGG16 network together with k-means clustering. Since in practice very few defective epoxy drop images are available on an actual IC production line, the emphasis in this paper is placed on the impact of data augmentation on the recognition outcome. The data augmentation is achieved by generating synthesized defective epoxy drop images via our previously developed enhanced loss function CycleGAN generative network. The experimental results indicate that when using data augmentation, the supervised and unsupervised models of VGG16 generate perfect or near perfect accuracies for recognition of defective epoxy drop images for the dataset examined. More specifically, for the supervised models of AE+MLP and VGG16, the recognition accuracy is improved by 47% and 1%, respectively, and for the unsupervised models of AE+Kmeans and VGG+Kmeans, the recognition accuracy is improved by 37% and 15%, respectively, due to the data augmentation.

## 1. Introduction

**Figure 1:**
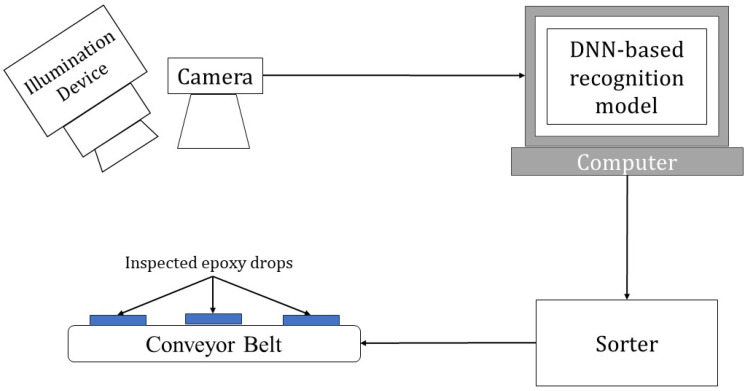
Workflow of a typical automated visual inspection system.

Integrated circuit (IC) manufacturing is a multi-stage and intricate process in which defects can be introduced at each stage [1]. Vision-based inspection systems are frequently used in IC manufacturing for the identification or recognition of defects. A typical vision-based inspection system consists of three main parts: a camera to capture images of interest, a computer to run a recognition module or software, and a sorter to separate defective from non-defective ICs. Deep neural network (DNN)-based classifiers are increasingly being used as the recognition module or software. Figure 1 depicts the workflow of a typical automated vision-based inspection system.

**Figure 2:**
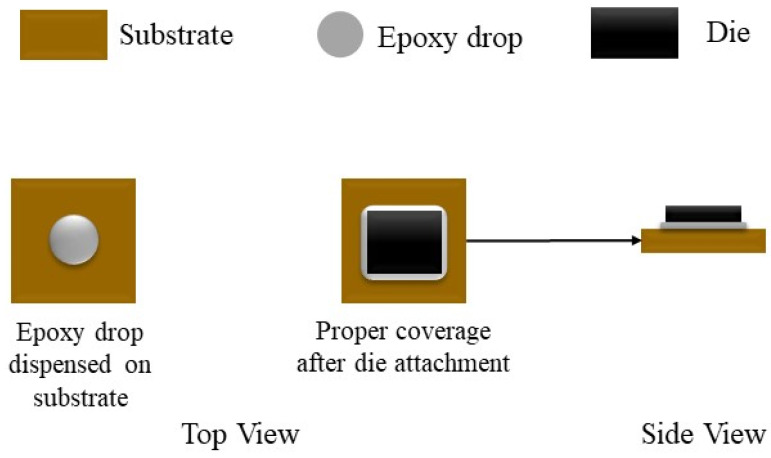
Die attachment via o-shape epoxy drop.

**Figure 3:**
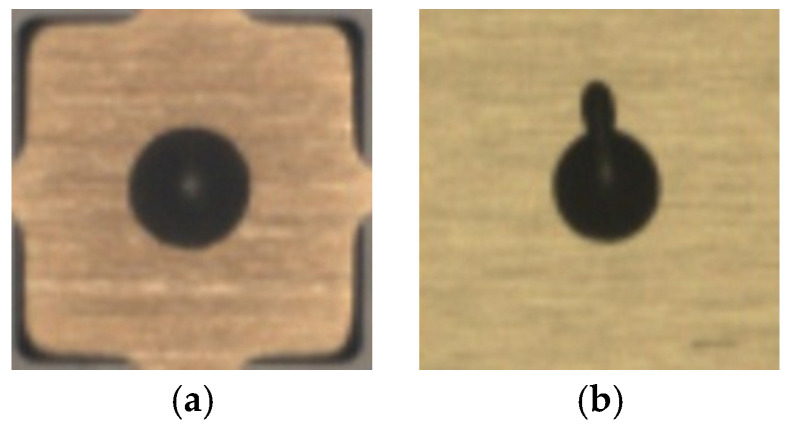
Sample images of epoxy drop: (**a**) good/non-defective, (**b**) defective.

Die attachment is an important stage in the production of ICs. Proper die attachment demands thermal/electrical efficiency and mechanical dependability. A commonly utilized die attachment technique involves using adhesive or epoxy [2]. Excessive or insufficient epoxy must be avoided as it leads to poor or defective die attachment. Therefore, an inspection is needed to identify defective epoxy drops placed on substrates to which dies get attached [3,4]. An illustration of die attachment is provided in Figure 2. A vision-based inspection system is used to identify dies that have an adequate amount of epoxy deposit. In other words, the epoxy deposit needs to be carried out such that a die neither tilts nor overflows its substrate, and the bond line maintains an optimal thickness. Dies that meet these criteria are labeled as non-defective or good and are passed through the inspection system without rejection. On the other hand, dies that exhibit excessive, inadequate and missing epoxy drop are labeled as defective and are rejected. Figure 3 shows a sample image of a non-defective or good epoxy drop image and a sample image of a defective or excessive epoxy drop image.

Most of the works in the literature have focused on identifying defective solder joints in printed circuit boards (PCBs), e.g., in [5,6,7,8] conventional machine learning techniques and in [9,10,11] deep learning networks were used for recognition of defective solder joints. Not much work has been reported in the literature on recognition of defective epoxy drops. In [12], conventional image processing techniques consisting of Canny edge detector and Hough transform were used to identify skewed, drained, and offset dies. In [13], seven machine learning approaches were examined for their ability to identify faults related to either an excessive or insufficient amount of glue on PCBs.

The design or training of a DNN-based classifier for the inspection of epoxy drop demands having a large number of image samples for both defective and non-defective cases. However, in practice, defective image samples occur rarely and thus an adequate number of defective epoxy drop images is not available for the design or training of a DNN-based classifier. To address the problem of not having an adequate number of defective epoxy drop image samples, data augmentation techniques can be utilized to generate synthesized defective image samples. For example, in [14], CycleGAN [15], a variant of generative adversarial network (GAN) [16,17], was used to generate realistic defective wafer maps. In this paper, defective epoxy drop images, which are generated by our previously developed improved CycleGAN generative network in [18], are used for data augmentation as part of a framework to recognize defective epoxy drop images.

Basically, the work reported in this paper examines the benefit of data augmentation for separating defective and non-defective cases. More specifically, both supervised and unsupervised recognition or classification are carried out to distinguish between defective and non-defective epoxy images. Two supervised models are considered which are (i) an autoencoder (AE) network [19] together with a multilayer perceptron (MLP) network [20] and (ii) a VGG16 network [21]. Furthermore, two unsupervised models are considered which are (i) an AE network together with k-means clustering [22] and (ii) a VGG16 network together with k-means clustering. The recognition outcomes are evaluated using widely used performance metrics.

The rest of the paper is organized as follows: Section 2 describes our recognition framework consisting of supervised and unsupervised models. The recognition results without and with data augmentation are then presented and discussed in Section 3. Finally, the paper is concluded in Section 4.

## 2. Methods

**Figure 4:**
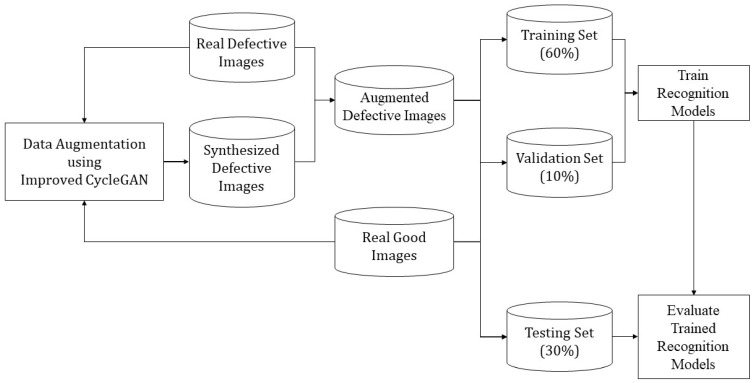
Setup of augmented or synthesized defective epoxy drop images for recognition models.

This section covers our recognition framework for identifying defective epoxy drop images in an automated way. The recognition is carried out both in a supervised and an unsupervised manner. Data augmentation plays a key role in the developed recognition framework. Figure 4 illustrates how synthesized images generated by data augmentation are used to set up training and testing sets for the recognition models presented later. As shown in this figure, after carrying out data augmentation of defective images, all of the samples of defective and good images are randomly divided with no overlap into 60% training, 10% validation, and 30% testing subsets.

### 2.1. Data Augmentation

In this paper, a dataset of o-shaped epoxy drop images from an IC manufacturer is examined to show the benefit of data augmentation. A predetermined region of interest (ROI) in the vision-based inspection system containing the epoxy is extracted which is of size 128 × 128 × 3 with 3 denoting the number of color channels. Among the images, only 16 epoxy drop images are labelled as defective while 8850 images are labelled as good or non-defective. Regardless of what recognition or classifier model is utilized, the rarity of the defective images as well as the imbalance number of samples between the defective and good cases would pose difficulties in reaching high recognition or classification accuracies. That is why it is essential to carry out data augmentation for this recognition problem.

The data augmentation technique we previously developed in [18] is used here. This technique is based on an improved CycleGAN generative network. Our improved CycleGAN involves an enhanced loss function. In [18], it was shown that by incorporating the measures of learned-perceptual-image-patch-similarity (LPIPS) and structural-similarity-index-metric (SSIM) into the standard CycleGAN loss function, more realistic or higher-quality synthesized epoxy drop images were generated. The CycleGAN optimization framework combines two losses: adversarial loss ($L_{advers}$) which measures the difference between generated images and target images and cycle consistency loss ($L_{cyc}$) which avoids conflicts between the learnt mappings. The total loss can be expressed as follows:

(1) $$L\left(G_{g\to d},G_{d\to g},D_{g},D_{d}\right)=L_{advers}\left(G_{g\to d},D_{d}\right)+L_{advers}\left(G_{d\to g},D_{g}\right)+L_{cycle}\left(G_{g\to d},G_{d\to g}\right)$$

where $G_{g\to d}$ & $G_{d\to g}$ denote the mapping functions for converting good images to defective images and vice versa, and $D_{d}$ & $D_{g}$ denote the associated adversarial discriminators. Cycle consistency loss is defined as the combination of forward ($L^{F\_Cycle}$) and backward $(L^{B\_Cycle}$) cycle consistency losses, that is

(2) $$L_{Cycle}\left(G_{g\to d},\;G_{d\to g}\right)=L^{F\_Cycle}\left(G_{g\to d}\right)+L^{B\_Cycle}\left(G_{d\to g}\right)$$

In our enhanced CycleGAN loss function, the following losses together with the standard CycleGAN *L*_1_ loss are used

(3) $$L^{F\_Cycle}\left(G_{g\to d}\right)=\alpha L_{L_{1}}^{F\_Cycle}+\beta L_{LPIPS}^{F\_Cycle}+\gamma L_{SSIM}^{F\_Cycle}\;$$

(4) $$L^{B\_Cycle}\left(G_{d\to g}\right)=\alpha L_{L_{1}}^{B\_Cycle}+\beta L_{LPIPS}^{B\_Cycle}+\gamma L_{SSIM}^{B\_Cycle}\;$$

with α, β, and γ denoting the weights assigned to each loss function. For more details, the reader is referred to [18].

The improved CycleGAN is thus used here to generate a dataset of defective epoxy drop images consisting of 1400 images by using a very small number of real defective epoxy drop images (16 of them). More synthesized images can be generated but 1400 images were found to be adequate for training and testing of the recognition models. Interested readers are referred to [18] for the details of this improved generative network and samples of synthesized defective images.

### 2.2. Supervised Recognition 

Two representative supervised recognition models of AE+MLP and VGG16 are utilized to show the benefit of the data augmentation in reaching high recognition accuracies. What is meant by supervised is that the training is conducted based on a labeled dataset. In other words, every training image sample is labelled as defective or good/non-defective by manual visual inspection.

#### 2.2.1. Autoencoder with Multilayer Perceptron 

The AE+MLP model which combines two networks of an autoencoder (AE) and a multilayer perceptron (MLP) is utilized as the first supervised model. The AE network performs feature extraction and the MLP network performs recognition or classification. The AE part provides a representation of images by a set of features. It consists of an encoder component which generates features from input images and a decoder component which reconstructs images from features. A fully connected (FC) layer with 64 units is used with the rectified linear unit (ReLU) activation function for the extraction of representative features by the encoder. After encoding, the decoder also consists of a FC layer with 49,152 units reconstructing the input image by using the sigmoid activation function. The loss function is set to mean squared error (MSE) and the Adam optimizer is used for training.

**Figure 5:**
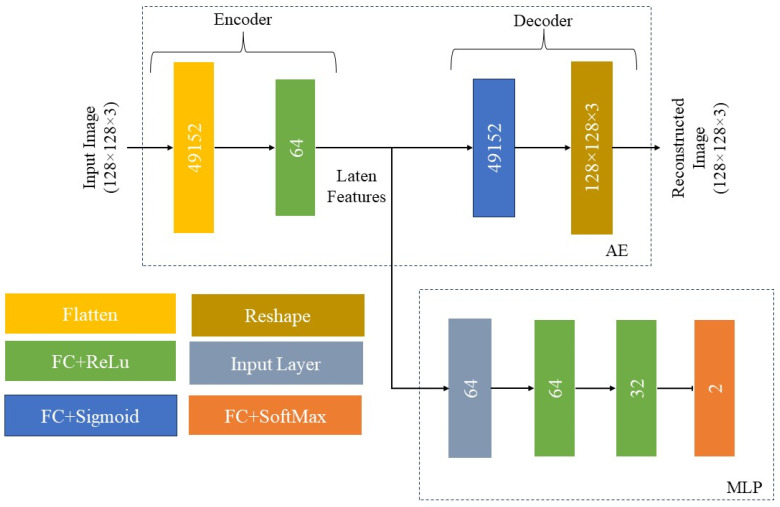
Architecture of the AE+MLP supervised recognition model.

After training the AE, the MLP is used to distinguish between defective and non-defective epoxy drop images by using the features extracted via the encoder. The MLP consists of two hidden FC layers with 64 and 32 units, respectively, with ReLU as the activation function, and an output FC layer with SoftMax as the activation function. The binary cross-entropy (BCE) loss function and the Adam optimizer are used for training of the MLP. Both of the networks are trained for 500 epochs determined by the validation set. The architectures of the two networks of this model are displayed in Figure 5.

#### 2.2.2. VGG16

**Figure 6:**
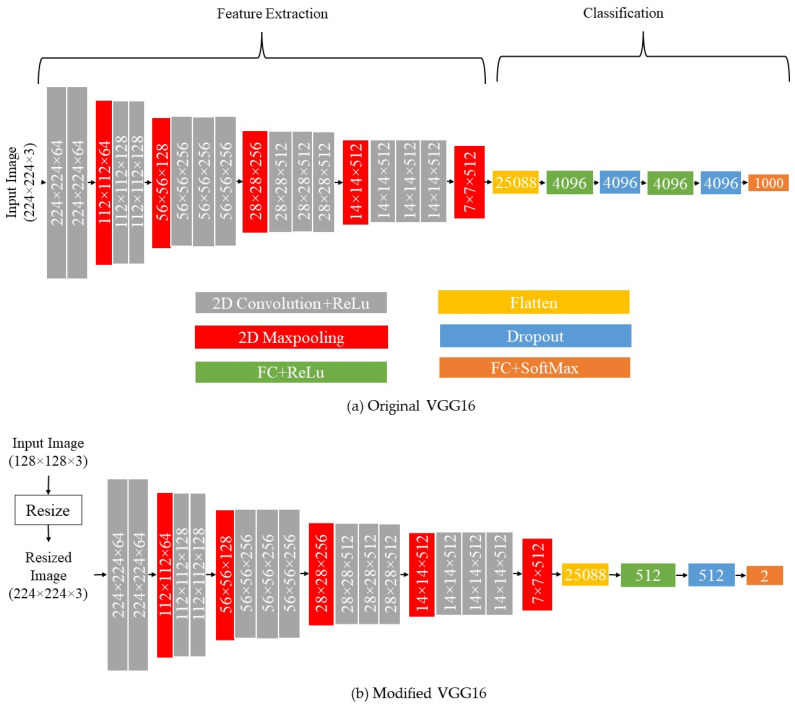
Architecture of the VGG16 supervised recognition model.

The pretrained VGG16 model is utilized as the second supervised model. The pretrained VGG16 is trained via more than a million images for one thousand classes in the ImageNet dataset. Only the output layer of this model is trained using the epoxy drop images. This model uses 16 convolutional layers with 3 by 3 filters. It can cope with 3 channel images with dimensions of 224 by 224. The last max-pooling layer in the model is linked to a 4096-unit FC layer, which is subsequently linked to a 1000 classification SoftMax layer as shown in Figure 6a. For our purposes, the top layers are excluded and replaced with a 512-unit FC layer and a SoftMax layer for our two-class recognition problem as shown in Figure 6b. Before the training is conducted, the images are resized since VGG-16 requires an input image size of 224 × 224. The model is trained for 500 epochs determined by the validation set. During the training, only the added custom layers to the VGG16 model are updated and all of the layers of the pretrained VGG16 model are frozen. The model is trained via the Adam optimizer based on the BCE loss function. 

### 2.3. Unsupervised Recognition

To take into consideration the situations when no manual visual inspection labeling of image data is carried out or available, two unsupervised recognition models are also considered in this work. In other words, the recognition is carried out without considering which images in the dataset correspond to good ones and which images to defective ones. K-means clustering is used once after the AE feature extraction and once after the VGG16 transfer learning to group or partition the epoxy drop images into two clusters of defective and good.

#### 2.3.1. Autoencoder with K-Means Clustering

**Figure 7:**
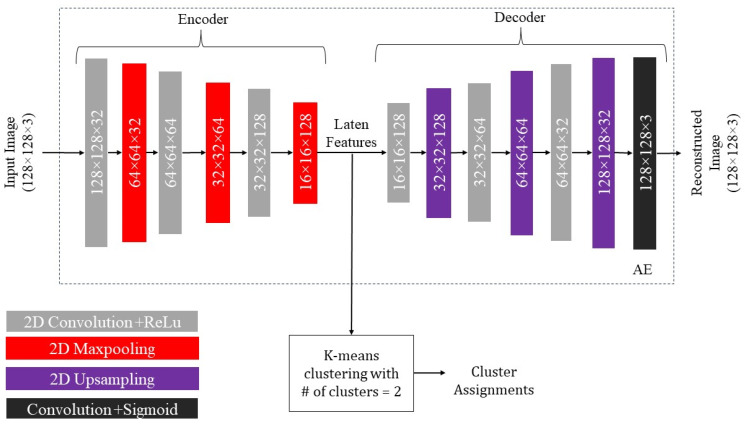
Architecture of the AE+Kmeans unsupervised recognition model (# means number).

Unlabeled training samples are used to train an AE that consists of an encoder and a decoder part. An input image regardless of its label is passed through the convolutional layers of the decoder with increasing filter sizes. After each convolutional layer, a 2 by 2 max-pooling is applied to downsample the spatial dimensions. The final output of the encoder or the last max-pooling layer form the input to k-means clustering. The encoded features are passed through the decoder consisting of a series of convolutional layers with decreasing filter sizes. After each convolutional layer, 2 by 2 upsampling is applied to increase the spatial dimensions. The ReLU activation function is used in the convolution layers except for the final layer which uses the sigmoid activation function. The training is carried out based on the MSE loss function together with the Adam optimizer. The AE network is trained for 1500 epochs determined by the validation set. Then, the output features of the encoder are used to perform k-means clustering. Figure 7 shows an illustration of the AE-based unsupervised model.

#### 2.3.2. VGG16 with K-Means Clustering

**Figure 8:**
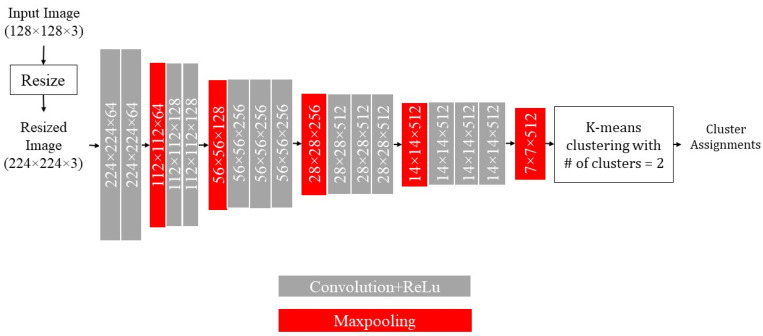
Architecture of the VGG16+Kmeans unsupervised recognition model (# means number).

Here the pretrained VGG16 is utilized for feature extraction before performing k-means clustering. As mentioned earlier, images with the dimensions of 224 × 224 × 3 need to be fed into the input layer, and the SoftMax layer provides 1000 output classes as illustrated in Figure 6a. The portion of the network labeled 7 × 7 × 512 from the input layer to the final max-pooling layer is considered to be the feature extraction portion of the model and the remaining portion is considered to be the classification portion of the model. Hence, for our purposes, the top layers are excluded (i.e., the FC and SoftMax layers) and only the convolutional and pooling layers are used for feature extraction. Using these pretrained layers, extracted features are then fed into k-means clustering to partition the unlabeled image samples. Figure 8 depicts the modified architecture of the VGG16-based unsupervised model. For visualization of clusters, principal component analysis (PCA) is applied to display the clusters using the two highest ranked principal components.

Four distinct recognition models are employed here, each with its own training. The AE+MLP model undergoes a sequential training process. It begins with an AE for initial feature extraction followed by a MLP for recognition. The VGG16 model, a pre-trained CNN, is fine-tuned for both feature extraction and recognition. In contrast, the AE+Kmeans model adopts a sequential training where an AE is used for feature extraction followed by k-means clustering for unsupervised recognition. Lastly, the VGG16+Kmeans model combines the VGG16 network with transfer learning for feature extraction and k-means clustering for unsupervised recognition. The unsupervised models provide an approach for defect recognition without relying on the availability of labeled data samples.

## 3. Results and Discussion

In this section, we report our recognition results for the two supervised and the two unsupervised models described above without and with data augmentation, i.e., without and with using synthesized defective images generated by our improved CycleGAN. All of the recognition models are implemented in Python using the TensorFlow and Keras libraries. Our experimentations were carried out on a server running 64-bit Windows 10 with two Intel Xeon 2.40 GHz CPUs and two 256 GB RAM NVIDIA Tesla K40m GPU boards.

### 3.1. Evaluation Metrics

The widely used evaluation metrics of precision, recall, F1-score, and accuracy of recognition models are reported here without and with the synthesized defective images. Table 1 presents the confusion matrix entries. From this matrix, the evaluation metrics of Recall, Precision, F1-score and Accuracy are computed as follows:

(5) $$Recall=\frac{TP}{TP+FN}$$

(6) $$Precision=\frac{TP}{TP+FP}$$

(7) $$F1-score=\frac{2\times Recall\times Precision}{Recall+Precision}$$

(8) $$Accuracy=\frac{TP+TN}{TP+TN+FP+FN}$$

where *TP* (true positive) indicates when a defective image is correctly placed in the defective class, *TN* (true negative) indicates when a good image is correctly placed in the good class, *FP* (false positive) indicates when a good image is incorrectly placed in the defective class, and *FN* (false negative) indicates when a defective image is incorrectly placed in the good class. To balance the samples from the two classes of good and defective, 1400 real good/non-defective images were selected randomly to match the number of synthesized defective images.

### 3.2. Visualization of Unsupervised Training Samples

**Figure 9:**
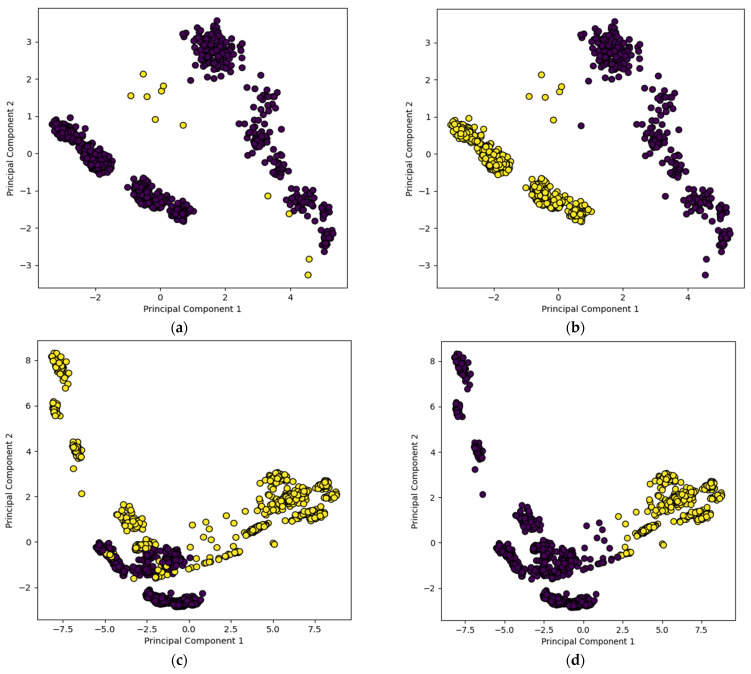
Visualization of two highest ranked principal components of the training samples using the unsupervised model of AE+Kmeans: (**a**) true labels without data augmentation, (**b**) assigned labels for (**a**), (**c**) true labels with data augmentation, and (**d**) assigned labels for (**c**).

**Figure 10:**
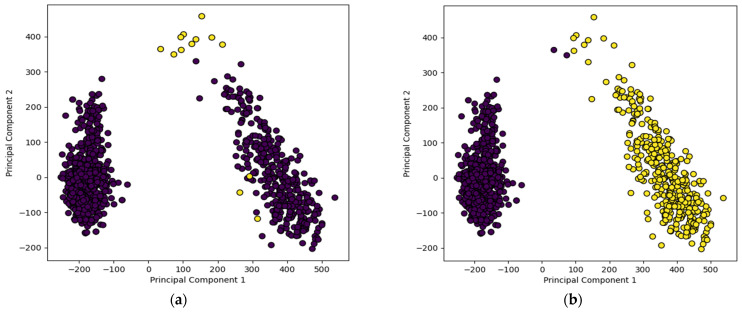

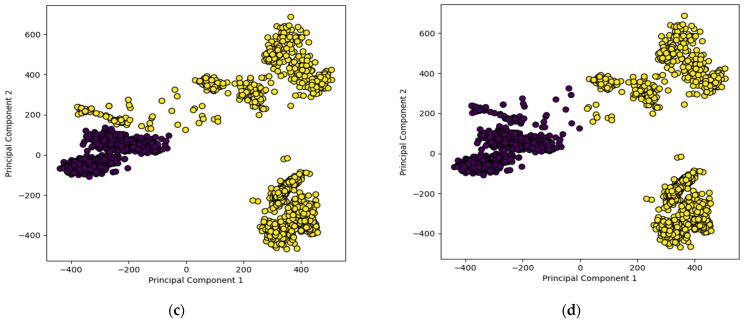
Visualization of two highest ranked principal components of the training samples using the unsupervised model of VGG16+Kmeans: (**a**) true labels without data augmentation, (**b**) assigned labels for (**a**), (**c**) true labels with data augmentation, and (**d**) assigned labels for (**c**).

Figure 9 and Figure 10 show the clustering of the training data using the unsupervised recognition models without and with data augmentation along with true or actual labels of the samples. Here, only the two highest ranked principal components are displayed for visualization purposes. Good/non-defective samples are represented by dark color circles and defective samples are represented by light color circles. Figure 9a,b show the true labels of the training samples and their clustering outcome by the AE+Kmeans unsupervised model without data augmentation, respectively, while Figure 9c,d show the true labels of the training samples and the clustering outcome by the AE+Kmeans unsupervised model with data augmentation, respectively. A similar set of figures are shown for the VGG16+Kmeans unsupervised model in Figure 10. As can be seen from these figures, the VGG16+Kmeans unsupervised model with data augmentation generated the best match with the true labels. More specifically, the AE-Kmeans unsupervised model produced a training accuracy of only 33% without data augmentation and a training accuracy of 78% with data augmentation; whereas the VGG16+Kmeans unsupervised model produced a training accuracy of 68% without data augmentation and a training accuracy of 97% with data augmentation.

### 3.3. Recognition Rates

Each of the recognition models were trained twice: once without data augmentation (i.e., by using only the real defective epoxy drop images) and then with data augmentation (i.e., by using the combination of the real and synthesized defective epoxy drop images that were generated by our enhanced loss function CycleGAN). In both cases good/non-defective images were kept the same. For the supervised recognition models, labelled image samples were used while for the unsupervised recognition models, the labels of the image samples were assumed to be unknown and were not used. Then, the trained models were tested by the same testing samples. The testing samples consisted of 420 good images and 425 defective images (real + synthesized).

Table 2 shows the recognition outcomes for the four models in terms of recall, precision, F1-score and accuracy. As can be seen from this table, the addition of the synthesized defective images improved the recognition outcome for both the supervised and unsupervised models. With data augmentation, it is seen that a perfect accuracy was obtained by the VGG16 supervised model and close to a perfect accuracy for the VGG16+Kmeans unsupervised model. Table 3 and Table 4 display the confusion matrices or recognition rates of the VGG16 supervised model and the VGG16+Kmeans unsupervised model with data augmentation, respectively.

In case of supervised recognition, although a high recognition rate was obtained using the VGG16 model without data augmentation, the addition of data augmentation provided a higher performance for both the VGG16 model and AE+MLP across all of the metrics of precision, recall, F1-score and accuracy. In case of unsupervised recognition, the performance of the models without data augmentation was poor while with data augmentation, the VGG16+Kmeans model achieved very high performance across all metrics. Hence, it is seen that data augmentation significantly improves the recognition performance in both supervised and unsupervised cases.

## 4. Conclusions

In this paper, both supervised and unsupervised recognition models were examined to show the benefit of data augmentation for achieving a more effective recognition or vision-based inspection of defective epoxy drop images in IC manufacturing. No previous papers in IC manufacturing have considered a generative network for data augmentation to conduct both supervised and unsupervised recognition. The supervised models considered were AE-MLP and VGG16 and the unsupervised models considered were AE and VGG16 together with k-mean clustering. In both cases, the models were trained without and with data augmentation using the same datasets. With data augmentation, the recognition results indicate that VGG16 as a supervised recognition model and VGG16 with k-means clustering as an unsupervised recognition model provide perfect or close to perfect recognition accuracies. More specifically, the data augmentation allowed improving the recognition accuracy of the VGG16 supervised model by 1% while it allowed improving the recognition accuracy of the VGG16+Kmeans unsupervised model by 15%. Finally, it is worth pointing out that the data augmentation recognition framework developed in this work is general purpose in the sense that it can be applied to other similar recognition problems. In our future work, it is planned to apply the same framework to other types of defects in the IC manufacturing process.

## Figures and Tables

**Table 1:** Confusion Matrix.

		Classified Labels	
		Defective	Good
Actual Labels	Defective	TP	FP
	Good	FN	TN

**Table 2:** Recognition rates for the supervised and unsupervised models.

	Recognition Model	Without Using Data Augmentation or Synthesized Defective Images				With Using Data Augmentation or Synthesized Defective Images			
		Recall	Precision	F1-Score	Accuracy	Recall	Precision	F1-Score	Accuracy
Supervised	AE+MLP	1.0	0.50	0.67	50%	0.99	0.96	0.97	97%
	VGG16	1.0	0.99	0.99	99%	1.00	1.00	1.00	100%
Unsupervised	AE+Kmeans	0.74	0.48	0.58	47%	0.72	1.00	0.84	86%
	VGG16+Kmeans	0.86	0.82	0.84	83%	0.97	1.00	0.98	98%

**Table 3:** Confusion matrix of the VGG16 supervised recognition model with data augmentation.

		Classified Labels	
		Defective	Good
Actual Labels	Defective	425	0
	Good	0	420

**Table 4:** Confusion matrix of the VGG16+Kmeans unsupervised recognition model with data augmentation.

		Classified Labels	
		Defective	Good
Actual Labels	Defective	411	14
	Good	0	420

## Data Availability

Data are contained within the article.
